# Translation, cultural adaptation, and psychometric testing of the measure for unfinished care among nursing assistants in long-term care homes in China

**DOI:** 10.3389/fpubh.2026.1829774

**Published:** 2026-04-16

**Authors:** Mengjiao Xu, Hongwei Qi, Yinfei Duan, Qiuyun Mao, Franziska Zúñiga, Yuting Song

**Affiliations:** 1 School of Nursing, Qingdao University, Qingdao, Shandong, China; 2 Faculty of Nursing, University of Alberta, Edmonton, AB, Canada; 3 Department of Nursing, Tai'an Central Hospital (Qingdao University Affiliated Tai'an Central Hospital & Taishan Geriatric Nursing Center), Tai'an, Shandong, China; 4 Department of Public Health, University of Basel, Basel, Switzerland; 5 Qingdao Municipal Key Laboratory for Smart Chronic Disease Management and Digital Rehabilitation Nursing, Qingdao, Shandong, China

**Keywords:** cross-cultural adaptation, long-term care homes, nursing assistants, psychometric testing, translation, unfinished care

## Abstract

**Objective:**

To translate and culturally adapt the Basel Extent of Rationing of Nursing Care for Nursing Homes (BERNCA-NH) into Chinese and to examine its preliminary psychometric properties among nursing assistants working in Chinese long-term care (LTC) homes for older adults.

**Methods:**

After obtaining permission from the original developers, the BERNCA-NH was translated and culturally adapted through forward translation, back-translation, synthesis, cognitive interviews, and expert review. A cross-sectional survey was conducted among 251 nursing assistants recruited by convenience sampling from long-term care facilities in China. Descriptive statistics, item analysis, exploratory factor analysis, and internal consistency testing were performed.

**Results:**

We identified three main challenges during the translation and cultural adaptation of the BERNCA-NH for use in Chinese LTC homes: syntactic and idiomatic differences between English and Chinese, mismatch between some original care scenarios and the local LTC context, and multiple possible Chinese interpretations of certain English expressions. The Chinese version of the BERNCA-NH showed good overall internal consistency (Cronbach’s *α* = 0.815). The KMO value was 0.746 and Bartlett’s test of sphericity was significant (χ^2^ = 1579.889, df = 210, *p* < 0.001), indicating suitability for factor analysis. Exploratory factor analysis supported a structure of five formal subscales and one single-item observation indicator, with a cumulative explained variance of 60.71%. The Cronbach’s *α* coefficients of the five subscales ranged from 0.622 to 0.764.

**Conclusion:**

The Chinese version of the BERNCA-NH showed acceptable preliminary psychometric properties and may serve as a preliminary tool for assessing unfinished care among nursing assistants in Chinese LTC homes, although further validation is needed. It may help identify priority areas for care improvement and provide support for quality assessment, management, and improvement in LTC homes.

## 1 Introduction

The quality of resident care provided in LTC homes remains a significant concern (1). Many facilities face persistent challenges, including resource shortages and frequent interruptions (2, 3). Unfinished care (also called care rationing, care left undone, unfinished care) commonly occurs when frontline staff workloads exceed available time and resources, leading staff to prioritize certain tasks, while others may be ignored, delayed, or reassigned (4–6). Most studies on unfinished care have been conducted in western countries. The limited research in Chinese LTC homes has reported that activities related to caring, rehabilitation, monitoring, documentation, and social support were frequently unfinished (7). Unfinished care is associated with multiple adverse health outcomes among residents (8), including urinary tract infections (9), avoidable hospitalizations (9), cardiovascular events (9), delirium (10), depression (11–13), functional decline (13), falls (14), urinary and fecal incontinence (15), nutrition problems (16), pain (13, 17), pressure ulcers (18), and even death (9, 19). Beyond its clinical risks, unfinished care substantially undermines residents’ quality of life. Residents reported that meeting their physical needs (e.g., via assistance from nursing assistants with bathing and dressing) was essential to maintaining meaning and purpose in life (20). They also regarded interaction with staff as central to their quality of life (21). When care aide interactions were interrupted because nursing assistants were urgently needed elsewhere, residents reported feeling uncomfortable and vulnerable (22). High-quality interactions with nursing assistants are essential for alleviating loneliness, a common issue among LTC residents (23, 24). These findings highlight the importance of studying and managing unfinished care for residents in LTC homes. However, unfinished care has rarely been studied in Chinese LTC homes, partly due to the lack of valid measures for the phenomenon.

Among available measurement tools for unfinished care (4, 25, 26), the Basel Extent of Rationing of Nursing Care in Nursing Home (BERNCA-NH) instrument has been translated into multiple languages, demonstrated reliability and validity, and widely applied among nursing assistants in international contexts (27–29). To the best of our knowledge, only one study (7) has used the BERNCA-NH instrument to measure unfinished care in Chinese LTC homes. However, the authors did not describe any process of translating or adapting the tool to the Chinese LTC home context. We were unable to reach the corresponding author for a Chinese version despite multiple attempts. Although care tasks for frontline staff are largely the same in LTC homes across countries, resident and staff characteristics differ across contexts. For example, nursing assistants in Chinese LTC homes often work extended shifts (7). Therefore, a culturally adapted Chinese version of a validated tool is warranted to evaluate unfinished care in Chinese LTC homes. In this study, we aimed to systematically translate and adapt the BERNCA-NH instrument for nursing assistants in Chinese LTC homes to ensure cultural adequacy and applicability.

## 2 Methods

### 2.1 The original BERNCA-NH instrument

The BERNCA-NH scale originates from the BERNCA (Basel Extent of Rationing of Nursing Care) instrument. The original BERNCA was designed to assess implicit rationing of nursing care in acute hospital settings, that is, situations in which registered nurses, owing to insufficient time, heavy workload, or limited resources, are unable to complete all essential nursing activities as required (28). It was subsequently adapted for nursing home settings as the BERNCA-NH, which measures the frequency with which care staff leave necessary care activities unfinished or only partially completed over the previous seven working days because of insufficient time or high workload. The original BERNCA-NH contains 19 items, applies to different categories of nursing home care staff, covers basic and routine care tasks, and comprises four dimensions: activities of daily living, care/rehabilitation and monitoring, documentation, and social care (27). The BERNCA-NH has since been translated, culturally adapted, and psychometrically evaluated in several countries, demonstrating promising cross-cultural applicability. Norman and Sjetne (30) developed and tested a Norwegian version, which resulted in four subscales: routine care, “when required” care, documentation, and psychosocial care. Later, Andersson et al. (31) extended the instrument to the Swedish community healthcare context and developed the BERNCA-NH/HC, a 29-item version with six subscales that further expanded its use in both LTC homes and home care settings. Nevertheless, variations across national versions in item content, dimensional structure, and care settings indicate that the Chinese translation and localization should not only retain the core concepts of the original instrument, but also reflect the specific care context, division of labor, and cultural background of Chinese LTC homes. Accordingly, the present study used the 21-item English adapted version of the BERNCA-NH provided directly by the Franziska Zúñiga team as the source instrument for Chinese translation and cultural adaptation.

### 2.2 Research design

This study was conducted in four phases, including (1) translation; (2) cultural adaptation of the Chinese Version of BERNCA-NH; (3) assessment of content validity; and (4) examination of the psychometric properties of the scale. Translation and adaptation of the BERNCA-NH scale were conducted with authorization from the original authors and under their guidance. We referenced general principles and methodological recommendations for cross-cultural translation and adaptation to ensure the scientific rigor and standardization of the Chinese version in linguistic expression, cultural appropriateness, and measurement properties.

#### 2.2.1 Phase 1: Translation

The translation process followed a systematic three-step approach: translation, back-translation, and synthesis. We followed established best practices using two pairs of independent translators and back-translators (32). ChatGPT 4.0 was used as one of the two translators in a pair in both processes (33). All prompts provided to ChatGPT4.0 are listed in Supplementary Table 1. The translation and cross-cultural adaptation procedures are summarized in Supplementary Figure 1.

##### 2.2.1.1 Initial translation

For this stage, a graduate student in nursing and ChatGPT 4.0 (configured as a graduate student majoring in English translation) independently translated the English scale into Chinese, producing two initial versions (T1 and T2). The translation was based on the standardized written English source version of the BERNCA-NH provided by the original developers. We also acknowledge that English varies across regions, registers, and spoken versus written forms. Accordingly, our aim was not literal word-for-word transfer, but preservation of the conceptual meaning of the source instrument within its original professional context. The goal of this initial translation was to preserve the original meaning of the English version as much as possible while aligning with the cultural and linguistic norms of Chinese. Simplicity and clarity were key considerations in word choice and phrasing. The research team then compared the two translations and resolved discrepancies through rounds of discussion and revision, producing the reconciled preliminary translation (T-final).

##### 2.2.1.2 Back translation

In this phase, a graduate student in nursing (blinded to the original scale) and ChatGPT 4.0 independently translated T-final back into English (34, 35). To improve the contextual alignment and task-specific accuracy of the back-translated version, we changed the role of ChatGPT 4.0 to a college English instructor with 10 years of experience in teaching and translation. This process yielded two back-translated versions (BT1 and BT2). The research team compared both back-translations with the original English version, discussed discrepancies, and agreed upon the reconciled back-translation (BT-final).

##### 2.2.1.3 Synthesis

The research team analyzed the original instrument, the preliminary translation (T-final), and the back-translated version (BT-final) side by side. This process generated the first draft of the Chinese version (Version 1).

#### 2.2.2 Phase 2: Cultural adaptation of the Chinese version of BERNCA-NH

To ensure alignment with the linguistic and cultural context of Chinese LTC homes, we followed a two-stage cross-cultural validation process to adapt the Chinese version of BERNCA-NH. This process involved cognitive debriefing with nursing assistants, followed by expert reviews.

##### 2.2.2.1 Cognitive debriefing

Cognitive debriefings were conducted with eight nursing assistants in one 95-bed LTC home located in urban Qingdao in northeastern China. Our research team has established long-term collaborations with this LTC home. To minimize potential conscious and unconscious bias related to this prior relationship, the interviews were conducted on a one-to-one basis, as far as possible in a private setting. Before each interview, participants were informed that the study was independent of the institution’s leadership and that their responses would not affect their work-related relationships. Trained graduate researchers explained the study purpose and provided clarification when needed to ensure participants understood the questionnaire items. Our inclusion criteria were: (1) current role as a care aide; (2) provision of direct care to older adults in the LTC home; and (3) at least six months of work experience in the current LTC home. We excluded part-time care providers and nursing assistants not directly engaged in older adult care. We introduced the study to the care manager of the home, who then recommended nursing assistants to us.

Following Willis’s cognitive interview method, five nursing assistants from the LTC home participated in a Think-Aloud exercise (36). Nursing assistants were asked about the scale’s clarity, applicability, and items they found difficult to understand. We identified and documented issues that nursing assistants reported.

Subsequently, three additional nursing assistants participated in follow-up interviews using the Verbal Probe method. They were asked targeted questions regarding their interpretation of survey items, the relevance of referenced scenarios to daily work, and the clarity and appropriateness of response options. Their feedback was recorded and analyzed to identify recurring issues. The interview outline and process are shown in Supplementary Table 2 and Supplementary Figure 2. Based on these insights, adjustments were made to produce a revised Chinese version of the scale (Version 2). Demographic information of participating nursing assistants is presented in Supplementary Table 3.

#### 2.2.3 Phase 3: Content validity assessment

The revised Chinese draft (Version 2), the back-translated version (BT-final), the original English scale, and rationale for any adjustment were sent to two external experts for evaluation. They are researchers with expertise in the unfinished care research field. The experts assessed each item for grammaticality, conceptual equivalence, and cultural adequacy. Their recommendations informed further refinement of the scale, particularly for items that were inaccurately translated, unclear, or culturally inconsistent, resulting in the final Chinese version (Version 3).

The final Chinese version (Version 3) incorporated both caregiver feedback and expert input, ensuring clarity, cultural adequacy, and suitability for use in Chinese LTC contexts.

#### 2.2.4 Phase 4: Examination of the psychometric properties of the scale

##### 2.2.4.1 Sample

This phase used a cross-sectional survey design and was conducted in 11 LTC homes in the cities of Qingdao and Tai’an in northeastern China. Participants were nursing assistants who were directly involved in providing daily care to older adults in LTC homes, and the inclusion criteria were consistent with those described in the cognitive debriefing section above. According to methodological recommendations for scale development and validation, the sample size for exploratory factor analysis is typically estimated as 5–10 participants per item, and a total sample size of 200–300 is generally considered adequate (37). A total of 272 questionnaires were collected in this study, of which 251 were valid after excluding invalid responses; therefore, the sample size was considered sufficient for the psychometric analysis of the scale.

##### 2.2.4.2 Data collection

Before the formal survey, the research team contacted administrators of the participating LTC homes in Qingdao and Tai’an to introduce the study objectives, content, and procedures, and field data collection was initiated after institutional permission had been obtained. Data were collected by members of the research team or trained survey investigators, who distributed paper-based questionnaires to nursing assistants meeting the inclusion criteria and provided on-site explanations regarding the study purpose, instructions for questionnaire completion, informed consent, and confidentiality requirements. The questionnaires were either completed and returned on site or completed independently within the specified time frame and then collected in a unified manner.

The survey questionnaire consisted of two parts. The first part collected general sociodemographic information, including gender, age, educational level, total years of caregiving experience, years of service at the current facility, shift pattern, average monthly income, number of assigned older adults, nursing assistant training, nursing assistant certification status, and turnover intention. The second part was the Chinese version of the BERNCA-NH scale. Data were collected from September 2024 to June 2025. To ensure data quality, researchers provided one-to-one explanation and clarification during the survey process and offered necessary assistance without influencing participants’ independent responses. After collection, all questionnaires were independently checked and verified by two researchers before being entered into the database for subsequent statistical analysis.

##### 2.2.4.3 Data analysis

Data were analyzed using IBM SPSS Statistics version 27.0. Descriptive statistics were used to summarize participant characteristics and item-level responses of the Chinese version of the BERNCA-NH. Categorical variables were described using frequencies and percentages. For item-level analysis, the frequency and percentage of responses in each category (“never,” “seldom,” “sometimes,” and “often”) during the last seven days were calculated for each item to examine the distribution of unfinished care activities.

Following the analytical approach used in the Swedish version of the BERNCA-NH/HC (31), exploratory factor analysis was conducted for items Q1–Q21 of the Chinese version of the scale. Exploratory factor analysis was selected because the present study aimed to provide an initial examination of the latent structure of the Chinese version within a single Chinese sample, rather than to test whether it replicated a prespecified factor structure of the source instrument. This approach was also considered appropriate because previous BERNCA-NH versions have shown some variation in dimensional structure across settings (30, 31), and several items required contextual adaptation in the Chinese LTC context. As all items in the dataset were recorded as four-point valid responses, with scores ranging from 1 to 4 representing “never” to “often,” all items were directly included in the analysis. Statistical analyses were performed using Python. The suitability of the data for factor analysis was first assessed using the Kaiser–Meyer–Olkin (KMO) test (38) and Bartlett’s test of sphericity (39). Principal component analysis was then applied to extract factors (40), followed by Oblimin oblique rotation with Kaiser normalization to interpret the factor structure (41). Factors with eigenvalues greater than 1 were retained (42), and each factor was named based on the cumulative variance explained and the item factor loadings. In general, a factor loading of ≥0.40 was considered an important criterion for item assignment, whereas items with cross-loadings were evaluated comprehensively in light of their content (40, 43). Finally, Cronbach’s *α* coefficient was used to assess the internal consistency of the total scale and each factor (44).

## 3 Results

### 3.1 Challenges with translation and cultural adaptation

We identified three types of challenges during the translation and cultural adaptation process, which are described below, with the corresponding strategies. Supplementary Table 4 illustrate examples of these challenges.

#### 3.1.1 Differences between English and Chinese versions regarding syntax and idiomaticity

During the translation and cultural adaptation process, the first challenge stemmed from fundamental differences in the syntactic structure between English and Chinese. English sentences usually start with the main clause or the clause core (subject, verb, and object), followed by modifiers (such as temporal, spatial, or conditional expressions), whereas Chinese typically places modifiers before the main clause or clause core (45, 46). As for the second challenge regarding the idiomaticity of lexical choices, literal translations of some English terms sounded overly formal or did not correspond to the daily language used by nursing assistants, creating a risk of misinterpretation (47). Therefore, literal translations often resulted in lengthy and awkward sentences that contradicted natural Chinese syntax and led to difficulties with comprehension for local nursing assistants.

As an example, Item 4 of the BERNCA Scale, “You could not provide patient(s) food if they are hungry at times other than the regular meals,” was initially literally translated as “如果机构里的老人在非正常用餐时间感到饥饿, 您无法提供食物给他们.” However, during cognitive interviews, nursing assistants reported that this phrasing was cumbersome and difficult to understand. After revisions, the phrasing was changed to: “在非正常用餐时间, 你未能给饥饿的老人提供食物” (Direct English translation: “During irregular mealtimes, you could not provide food to older adults who were hungry”). The revised version places the time-related phrase (“during irregular mealtimes”) at the beginning, which made the sentence clearer and easier for nursing assistants to understand in the questionnaire context.

Similarly, Item 5 stated: “You were unable to mobilize residents or change the position of residents, who had restricted mobility/motility or who were immobile.” The initial translation was: “你无法使老人自己移动或改变行动受限/行动不便或无法行动的机构老人的位置.” However, nursing assistants reported that this phrasing was difficult to understand and lacked clarity. We subsequently revised it to: “对于那些行动受限或完全不能活动的老人，你未能移动老人或者改变老人的姿势.” (Literal English translation: “For older adults individuals with limited mobility or who are completely immobile, you were unable to move the older adults person or adjust their position.”) Our revision places the subject of the sentence (“older adults individuals with limited mobility”) at the beginning and uses more common, concrete expressions, like “move the older adults person” and “adjust their position,” significantly improving readability for nursing assistants.

Another example was Item 6: “You have to expose an older person in a facility to urine or feces for an extended period of time (more than 30 min).” The initial translation was: “您不得不让机构里的老人长时间(超过30分钟)留在尿液或粪便中.” Caregivers indicated that this phrasing was overly complex. We revised the item to: “你不得不让老人的尿液或粪便留在身上超过 30 分钟.” (Literal English translation: “You must not allow an older adults person’s urine or feces to remain on their body for more than 30 min.”) This version replaces “remain in” with “stay on their body,” which more directly reflects the care situation and nursing assistants described easier to understand during cognitive interviews.

Through these adjustments in syntax and wording, we sought to preserve the intended meaning of the original items while improving clarity, contextual appropriateness, and comprehensibility for Chinese nursing assistants in LTC homes.

#### 3.1.2 The scenarios mentioned in the original scale did not fit the context in local LTC homes

Several scenarios in the original BERNCA-NH did not align with daily practices in Chinese LTC homes. In cognitive interviews, nursing assistants reported that items such as “go for a walk,” “shopping,” “cooking,” “excursion,” “visit to the city,” and “activities with children” were uncommon in their care homes. We asked for suggestions from participating nursing assistants and consulted the original developer team of BERNCA-NH. We later revised these items to reflect scenarios more common in Chinese LTC homes. For example, “go for a walk” was adapted to “机构内散步” (walking within the facility), “shopping” to “机构外买东西” (buying items outside the facility), and “cooking” to “机构内做操” (exercise within the facility). In the Chinese LTC context, cooking-related activities are uncommon in routine institutional practice because they usually require closer supervision and additional staff support to ensure safety. These revisions were intended to improve contextual relevance, although some adaptations may not fully preserve all of the social and cultural meanings embedded in the original examples.

#### 3.1.3 Different English expressions had multiple interpretations in Chinese

Challenges arose from some English expressions having different interpretations when translated into Chinese. Careful judgment was required to select terms that conveyed the intended meaning accurately in context. During the translation of the BERNCA-NH scale from the original English to Chinese, the scale entries “could not” and “unable to” were translated as “未能(failed)” and “无法(unable to),” respectively. However, the expert reviewers clarified that both English phrases were used in the original as having the same basic meaning, while the two Chinese expressions differ with respect to meaning and formality. In Chinese, “未能” indicates that a goal was not achieved due to unfavorable conditions or inadequate circumstances. It emphasizes a person’s or thing’s failure to achieve a desired result or accomplish a task in a given situation.”未能” is commonly used in spoken Chinese, such as daily conversation, informal writing, or oral reports. On the other hand, “无法” indicates an inability to fulfill a requirement. It emphasizes lacking the ability or conditions to perform a task or achieve a goal. It is used more often in formal contexts or written language, such as official documents, reports, or statements. After consulting with the expert reviewers, “无法” was selected as more appropriate in all cases.

The aforementioned revisions ensured the translations conveyed the meaning of the original scales while remaining clear and appropriate for Chinese nursing assistants.

### 3.2 Psychometric assessment of the Chinese version of the BERNCA-NH

A total of 251 nursing assistants from LTC homes participated in the examination of the psychometric properties of the scale. Participants were predominantly female (81.7%), and the largest age group was 45–54 years (41.8%). Most participants had a junior high school or senior high school education (60.2%), 2–5 years of total caregiving work experience (49.0%), and 3 years or more of service at their current facility (43.8%). Additional demographic characteristics are presented in Table 1.

**Table 1:** Demographics of participating nursing assistants.

Variables	Frequency (*n*)	Percentage (%)
Gender		
Male	46	18.3
Female	205	81.7
Age		
≤25 years	33	13.1
25–44 years	54	21.5
45–54 years	105	41.8
≥55 years	59	23.5
Educational level		
Primary school or below	24	9.6
Junior high school or senior high school	151	60.2
Associate degree or above	76	30.3
Total caregiving work experience		
≤2 years	65	25.9
2–5 years	123	49.0
≥5 years	63	25.1
Years of service at current facility		
6 months–1 year	45	17.9
1–2 years	54	21.5
2–3 years	42	16.7
≥3 years	110	43.8
Shift worked most of the time		
Day shift	26	10.4
Rotating two-shift system	87	34.7
Continuous 24-h duty shifts	115	45.8
Other	23	9.2
Average monthly income		
≤3,000 CNY	21	8.4
3,001–4,000 CNY	67	26.7
4,001–4,999 CNY	84	33.5
≥5,000 CNY	79	31.5
Number of older adults cared for per shift		
≤6	64	25.5
7–9	77	30.7
≥10	110	43.8
Received nursing training		
Yes	249	99.2
No	2	0.8
Turnover intention		
Within 6 months	8	3.2
Within 1 year	19	7.6
No plan to leave	224	89.2

Item analysis showed that the frequency of unfinished care varied across activities. The least frequently unfinished activities were Change/apply wound dressings, Administer medication, and Respond to residents who ring, whereas the highest proportions in the “Often” category were observed for Emotional support, Communicate with resident/family, and Toileting/continence training. Response distributions and percentages for all items are presented in Table 2.

**Table 2:** Nursing assistants’ descriptive and item response distributions (%).

Unfinished care activities reported for the last seven days	Never	Seldom	Sometimes	Often
Sponge bath/skin care	215 (85.7)	31 (12.4)	4 (1.6)	1 (0.4)
Oral hygiene	214 (85.3)	27 (10.8)	7 (2.8)	3 (1.2)
Assist eating/drinking	225 (89.6)	18 (7.2)	6 (2.4)	2 (0.8)
Provide extra meals	219 (87.3)	22 (8.8)	5 (2.0)	5 (2.0)
Mobilization/position change	228 (90.8)	18 (7.2)	4 (1.6)	1 (0.4)
Clean excreta promptly	233 (92.8)	15 (6.0)	2 (0.8)	1 (0.4)
Emotional support	162 (64.5)	64 (25.5)	14 (5.6)	11 (4.4)
Communicate with resident/family	166 (66.1)	49 (19.5)	25 (10.0)	11 (4.4)
Toileting/continence training	182 (72.5)	37 (14.7)	22 (8.8)	10 (4.0)
Rehabilitative/activating care	204 (81.3)	29 (11.6)	12 (4.8)	6 (2.4)
Monitor residents	202 (80.5)	29 (11.6)	16 (6.4)	4 (1.6)
Monitor cognitively impaired	160 (63.7)	52 (20.7)	32 (12.7)	7 (2.8)
Respond to residents who ring	233 (92.8)	17 (6.8)	1 (0.4)	0 (0)
Administer medication	244 (97.2)	7 (2.8)	0 (0)	0 (0)
Change/apply wound dressings	249 (99.2)	1 (0.4)	1 (0.4)	0 (0)
Review care plan	203 (80.9)	28 (11.2)	13 (5.2)	7 (2.8)
Set/update care plan	210 (83.7)	28 (11.2)	9 (3.6)	4 (1.6)
Document care	201 (80.1)	28 (11.2)	19 (7.6)	3 (1.2)
Individual activity	178 (70.9)	49 (19.5)	17 (6.8)	7 (2.8)
Group activity	214 (85.3)	27 (10.8)	9 (3.6)	1 (0.4)
External cultural activity	207 (82.5)	23 (9.2)	15 (6.0)	6 (2.4)

The KMO measure of sampling adequacy was 0.746, and Bartlett’s test of sphericity was significant (χ^2^ = 1579.889, df = 210, *p* < 0.001), indicating that the data were suitable for factor analysis. Principal component analysis with Oblimin rotation identified six factors with eigenvalues greater than 1.0, accounting for 22.98, 10.78, 8.91, 6.40, 5.84, and 5.80% of the variance, respectively, with a cumulative explained variance of 60.71%. The factor loadings of the items and the names of the subscales are presented in Tables 3, 4. It should be noted that item 15 (“Change/apply wound dressings”) showed a distinctive statistical pattern in this study. Of the 251 respondents, 249 selected the lowest response category, and only 2 selected other categories, indicating a highly concentrated response distribution and extremely low item variability. Although item 15 loaded as a single-item factor in the exploratory factor analysis, this finding more likely reflects its weak association with the other items rather than a stable and independent latent construct. From a content perspective, wound dressing care is a relatively technical and role-specific task, which may not be routinely performed by all nursing assistants in LTC homes. Therefore, item 15 was not retained as a formal subscale in the final factor structure, but was instead kept as a single-item observation indicator.

**Table 3:** Pattern matrix with factor loadings; Basel Extent of Rationing of Nursing Care for Nursing Homes, Chinese version.

Subscales and items	I	II	III	IV	V	VI
Basic life care						
Sponge bath/skin care	**0.727**	0.090	0.082	0.030	0.137	0.084
Oral hygiene	**0.754**	0.123	0.065	0.092	0.116	0.316
Assist eating/drinking	**0.534**	0.131	**0.345**	0.250	0.110	0.174
Provide extra meals	**0.527**	0.254	0.121	0.020	0.262	0.236
Mobilization/position change	**0.566**	0.223	0.064	0.257	0.037	0.123
Care responsiveness and documentation						
Clean excreta promptly	0.330	**0.504**	0.030	0.007	0.045	0.055
Respond to residents who ring	0.030	**0.673**	0.035	0.014	0.246	0.325
Administer medication	0.010	**0.547**	0.075	0.134	0.260	0.107
Review care plan	0.010	**0.643**	0.253	0.241	0.071	0.022
Document care	0.037	**0.568**	0.258	0.202	0.069	0.106
Dignity, support, and condition monitoring						
Emotional support	0.023	0.153	**0.776**	0.033	0.090	0.040
Communicate with resident/family	0.061	0.042	**0.745**	0.192	0.102	0.005
Monitor residents	0.055	0.066	**0.508**	0.307	0.078	0.108
Monitor cognitively impaired	0.040	0.189	**0.737**	0.039	0.310	0.218
Routine care promotion and care planning						
Toileting/continence training	0.007	0.073	0.234	**0.744**	0.069	0.138
Rehabilitative/activating care	0.119	0.127	0.022	**0.802**	0.077	0.055
Set/update care plan	0.074	0.352	0.087	**0.675**	0.041	0.178
Social activities and participation						
Individual activity	0.029	0.005	0.057	0.322	**0.656**	0.130
Group activity	0.007	0.298	0.026	0.098	**0.745**	0.046
External cultural activity	0.155	0.050	0.020	0.017	**0.739**	0.080
Single-item observation item						
Change/apply wound dressings	0.049	0.119	0.048	0.025	0.136	**0.889**

Bold values indicate the salient factor loading(s) for each item. These values were used to determine item grouping into subscales.

**Table 4:** Overview of subscales, number of items, Cronbach’s alpha.

Factor/Subscale	Label	No. of items	Cronbach’s α
Factor I	Basic life care	5	0.666
Factor II	Care responsiveness and documentation	5	0.689
Factor III	Dignity, support, and condition monitoring	4	0.764
Factor IV	Routine care promotion and care planning	3	0.739
Factor V	Social activities and participation	3	0.622
Factor VI	Single-item observation item (change/apply wound dressings)	1	—

After excluding Q15 from interpretation as an independent dimension, the remaining items clustered into several conceptually meaningful subscales, including Basic life care, Care responsiveness and documentation, Dignity, support, and condition monitoring, Routine care promotion and care planning, and Social activities and participation. These subscales broadly captured the major domains of unfinished care among nursing assistants in LTC homes. The overall Cronbach’s alpha of the scale was 0.815, indicating good internal consistency of the Chinese version of the BERNCA-NH.

## 4 Discussion

### 4.1 Challenges and implications of translation and cultural adaptation

During the translation and cultural adaptation process, we encountered challenges similar to those reported in previous scale translation studies, as well as unique issues specific to this study. Similar to prior studies, we addressed differences in syntax, idiomaticity, and local context (48, 49). Structurally, English sentences are often longer, rely on passive voice, and use post-modifying elements. In contrast, Chinese sentences are generally more concise, favor active verbs, and use pre-modifying elements (50). Accordingly, we made careful structural adjustments during translation to ensure clarity and readability of the Chinese version. From a linguistic perspective, these adjustments reflect systematic differences in constituent order between English and Chinese. While both languages show a basic subject-verb-object clause structure, idiomatic Chinese sentences often favor topic-comment structures (with fronted constituents like objects, temporal expressions or the like) (51).

We also faced acculturation challenges rarely reported in previous studies, particularly regarding scenario-specific adaptations in LTC homes. The BERNCA-NH scale was originally developed in European and American contexts. Participating nursing assistants reported that many of the referenced scenarios (e.g., cooking, shopping) were uncommon in their daily practices in Chinese LTC homes and therefore required cultural adaptation. Also, certain aspects of daily care (e.g., handcrafting, doing aerobics) in the scales were uncommon in Chinese LTC homes and thus required modification. For example, older adults in LTC homes in Sweden, Germany, and Norway typically have more opportunities for outdoor activities than residents in Chinese LTC homes, leading to fewer acculturation issues during scale translation among the European languages (30, 31). These adaptations improved contextual relevance in Chinese LTC homes. However, they may not have fully preserved the broader social and cultural meanings of the original examples.

The findings of this study provide an important foundation for future research on cross-cultural adaptation of measurement tools, particularly for the Chinese LTC context. This study explored integrating human and machine translation, employing ChatGPT 4.0 to enhance both efficiency and accuracy in translation. Although machine translation can produce rapid drafts, manual refinement remains essential to ensure cultural relevance. Future studies should explore optimal strategies for integrating artificial intelligence tools with human input to strengthen cross-cultural adaptation processes. The optimized Chinese version of the BERNCA-NH scale developed in this study potentially provides a reliable tool to assess unfinished care in Chinese LTC homes. We are collecting data to assess the reliability and validity of this tool.

### 4.2 Psychometric properties and multidimensional structure of the Chinese version of the BERNCA-NH

The psychometric assessment showed good overall internal consistency. Considering both item content and factor structure, the current version is best interpreted as comprising five formal subscales and one single-item observation indicator. This suggests that unfinished care in LTC homes is multidimensional, involving basic life care, timely response, documentation and implementation, functional promotion, social participation, and relational care.

The original BERNCA-NH yielded four subscales (27), and the Norwegian version likewise identified four subscales (30), whereas the Swedish version, based on an expanded item set, produced six subscales (31). Therefore, although the present findings show good conceptual continuity with previous versions, they differ somewhat in the allocation of specific items and the number of subscales. This suggests that the factor structure of the instrument is shaped not only by the original theoretical framework, but also by differences in LTC systems, institutional management models, and role delineation across countries.

The handling of item 15 (“Change/apply wound dressings”) was a key issue in this study. Of the 251 respondents, 249 selected the lowest response category, and only 2 selected other categories, indicating extremely low variability. Although Q15 emerged as a single-item factor in the exploratory factor analysis, this finding is more likely to reflect its weak association with the other items than a stable and independent latent construct (52). From a content perspective, wound dressing care is a relatively technical activity that requires a higher level of professional judgment. In LTC homes, it often involves clearer competency boundaries, documentation requirements, and support from care planning; therefore, its strong role specificity among nursing assistants is understandable (53, 54). On this basis, retaining item 15 as a single-item observation indicator, rather than including it in the formal subscale structure, appears to be a more prudent approach that balances statistical results with content validity. Future studies may further evaluate how this item should be retained and interpreted in relation to job category, role delineation, and actual workflow. The five formal subscales identified in this study showed good content interpretability, further indicating that unfinished care in LTC homes is clearly multidimensional rather than simply reflecting omissions of isolated care tasks (27, 55). Among these, Basic life care included sponge bath/skin care, oral hygiene, assist eating/drinking, provide extra meals, and mobilization/position change, all of which represent the most direct and fundamental physical care tasks performed by nursing assistants. These items are all related to the maintenance of activities of daily living (ADL), which provides a strong practical basis for their clustering into a relatively independent subscale. Previous BERNCA-NH studies have likewise shown that basic care is one of the most stable core domains of unfinished care in LTC homes (27, 30). In addition, LTC research has demonstrated that functional maintenance and ADL support are closely associated with older adults’ physical status, level of dependence, and quality of life. Therefore, this subscale reflects not only task completion, but also a core aspect of the quality of basic care in LTC homes (27, 56).

The Care responsiveness and documentation subscale included timely excreta care, responding to residents’ calls, administering medication on time, reviewing care plans, and documenting care provided. Although these tasks may appear scattered, they all involve the recognition of residents’ immediate needs and the extent to which care delivery can be followed through and accurately documented. Reviews of documentation and care planning in LTC homes have shown that documentation quality, care plan updating, information transfer, and person-centred care are closely interconnected, and that electronic health records and nursing documentation systems directly affect assessment, care planning, information exchange, and accountability (57, 58). Therefore, the clustering of these items into one subscale may reflect the continuous chain of response–implementation–documentation in the actual workflow of nursing assistants in Chinese LTC homes.

The Dignity, support, and condition monitoring subscale included emotional support, communication with residents or family members, close monitoring of residents, and observation of cognitively impaired residents, suggesting a strong conceptual linkage among companionship, dignity preservation, and observational care. Relational care and psychosocial support are often left unfinished in higher rates than task-oriented activities, as also supported by our study (31, 59, 60). Previous studies have shown that person-centred care emphasizes residents’ relationships, preferences, life histories, and family involvement, while family communication, care planning, and documentation are also essential components of person-centred care (61, 62). Therefore, grouping these items into one subscale appears to be both theoretically and practically justified.

The Routine care promotion and care planning and Social activities and participation subscales further extend the understanding of unfinished care. The former highlights toileting/continence training, promotion of independence, and updating care plans, suggesting that care in LTC homes involves not only completing immediate tasks but also supporting residents’ functional maintenance and future care arrangements. The latter indicates that individual activities, group activities, and opportunities for outside contact are not optional extras, but important aspects of residents’ participation, social connection, and sense of meaning (58, 63). Reviews of activity interventions in LTC homes have shown that individualized, enjoyable, and meaningful activity participation is closely related to residents’ well-being, and quality improvement research in LTC has likewise emphasized that participation and quality of life should not be treated as peripheral concerns (63, 64).

The overall Cronbach’s alpha of the Chinese version of the BERNCA-NH was 0.815, indicating good internal consistency and supporting its preliminary use. The five formal subscales showed alpha coefficients ranging from 0.622 to 0.764, suggesting some variation in internal homogeneity. In particular, Dignity, support, and condition monitoring and Routine care promotion and care planning performed relatively better, whereas social activities and participation was lower. This finding should be interpreted cautiously, as some subscales contained few items and others encompassed relatively heterogeneous tasks that may be influenced by institutional resources, role allocation, and shift arrangements. Methodological studies have shown that Cronbach’s alpha is affected not only by inter-item correlations but also by the number of items and the homogeneity of the construct (65, 66). In addition, systematic reviews of unfinished nursing care instruments have shown that variation in subscale reliability across versions and settings is common (6, 59). Consequently, the present findings support the preliminary applicability of the Chinese version, although the stability of some subscales requires further testing in larger and more diverse samples.

### 4.3 Practical and public health implications

The Chinese version of the BERNCA-NH may have practical and public health value in LTC homes. In practice, it may help care teams and managers identify which aspects of daily care are most often left unfinished and where improvements in staffing, workflow, training, or care organization may be needed. Existing studies in LTC settings suggest that unfinished care is measurable, occurs frequently, and is associated with modifiable features of the care environment, indicating that such assessments may be useful for quality monitoring and service improvement (5, 6, 30, 67).

Also, because unfinished care is associated with multiple resident outcomes (20–24), it can serve as a signal of whether older adults’ daily needs are being met in a timely, consistent, and respectful way. Research on person-centred care in LTC shows that dignity, autonomy, relationships, and attention to individual needs are closely linked to residents’ quality of life and well-being (68, 69). Accordingly, identifying unfinished care may help LTC homes better recognize gaps that affect residents’ comfort, dignity, and quality of life, rather than focusing only on task completion.

Over time, the instrument may also provide a useful way to evaluate whether efforts to improve care delivery are leading to more complete care and better resident-centred outcomes. In addition, because staffing and skill mix have been associated with resident outcomes in LTC homes, findings from this tool may also help support local discussions about staffing, training, and care priorities in Chinese LTC homes (70). Further qualitative research would be valuable for understanding how unfinished care is experienced and addressed by nursing assistants and residents in everyday LTC practice.

### 4.4 Limitations

Several limitations should be acknowledged. First, participants were recruited from LTC homes in urban areas in northeastern China, which may limit the representativeness and generalizability of the findings. The applicability and stability of the scale structure among nursing assistants with different characteristics from our sample require further examination (59, 71). Second, exploratory factor analysis was used to provide an initial examination of the latent structure of the Chinese version of the BERNCA-NH, but this is insufficient to definitively confirm the factor structure. Further validation using confirmatory factor analysis is needed (72). In addition, Item 15 (“Change/apply wound dressings”) showed clear role specificity and very low variability, suggesting that some items may not apply to our participants. Future studies should further evaluate the retention, scoring, and structural placement of such items. Finally, the BERNCA-NH was originally developed in LTC contexts that differ from those in China, and some items may therefore remain context-specific despite cultural adaptation, which may limit comparability and generalizability. Moreover, the Chinese translation in this study was based on the 21-item English adapted version provided by the Franziska Zúñiga team rather than directly on the earliest original-language version of the instrument. This should also be considered when interpreting the translation and cultural adaptation process.

## 5 Conclusion

This study translated and culturally adapted the BERNCA-NH for use in Chinese LTC homes through a rigorous process involving forward–backward translation, cognitive debriefing, and expert review. The Chinese version demonstrated acceptable preliminary psychometric properties and may serve as a feasible instrument for assessing unfinished care among nursing assistants in Chinese LTC homes. Beyond its research use, it may help care teams and managers identify gaps in daily care, support care quality monitoring, quality improvement, and management in LTC homes. In the longer term, its use may contribute to more complete care, better care quality, and improved resident well-being and quality of life. Further studies are needed to evaluate its psychometric properties in larger and more diverse samples.

## Data Availability

The data supporting the conclusions of this article are available from the corresponding author, YS, upon reasonable request.
