# Supplementary material for: Translation, cultural adaptation, and psychometric testing of the measure for unfinished care among nursing assistants in long-term care homes in China
Source: Front Public Health. 2026 Apr 16;14:1829774. doi: 10.3389/fpubh.2026.1829774 (PMC13130219; doi:10.3389/fpubh.2026.1829774)

Supplementary figure 1. Flowchart of the translation and cross-cultural adaptation process of developing the Chinese version of the Basel Extent of Rationing of Nursing Care for LTC home instrument


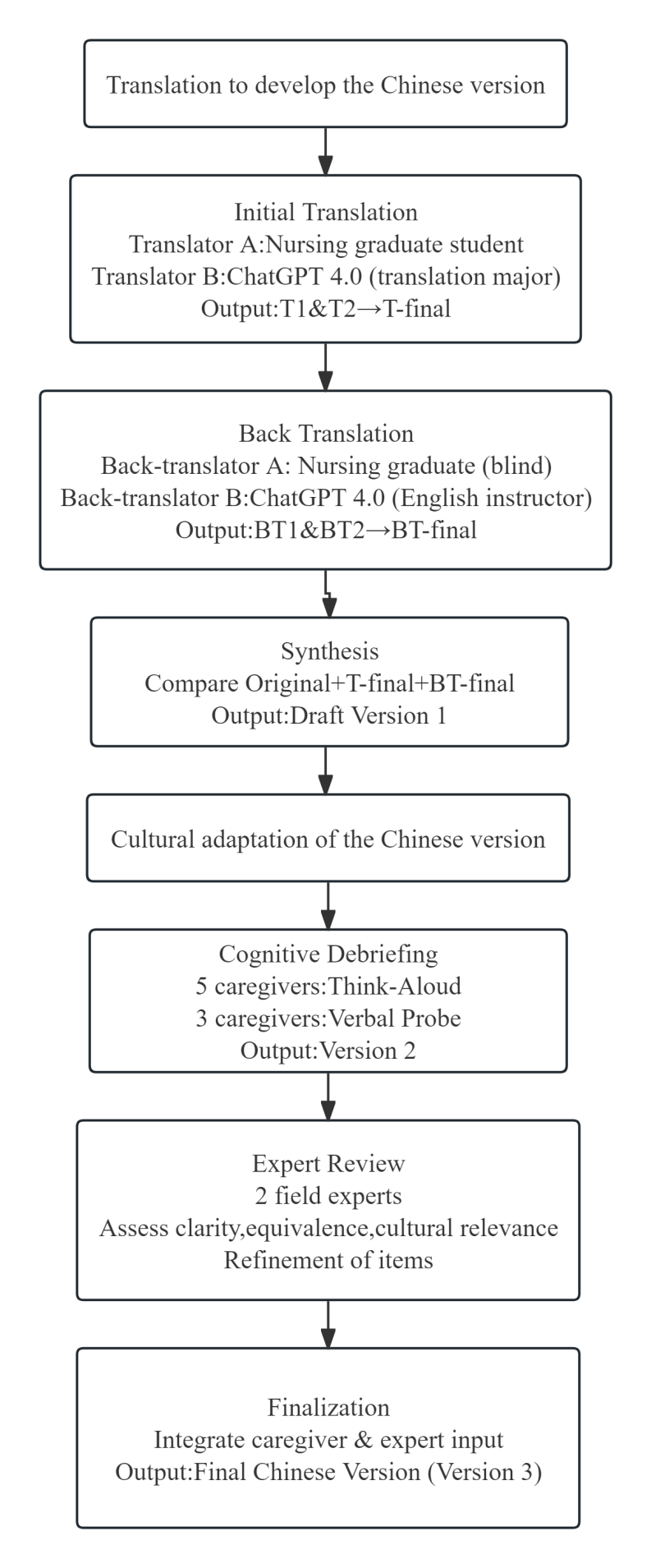

Supplement: Supplementary file 1 [file Supplementary_File_1.docx]
