# Supplementary material for: Translation, cultural adaptation, and psychometric testing of the measure for unfinished care among nursing assistants in long-term care homes in China
Source: Front Public Health. 2026 Apr 16;14:1829774. doi: 10.3389/fpubh.2026.1829774 (PMC13130219; doi:10.3389/fpubh.2026.1829774)

Supplementary figure2. Procedure of cognitive debriefing used in this study for

developing the Chinese version of the Basel Extent of Rationing of Nursing Care for LTC homes instrument


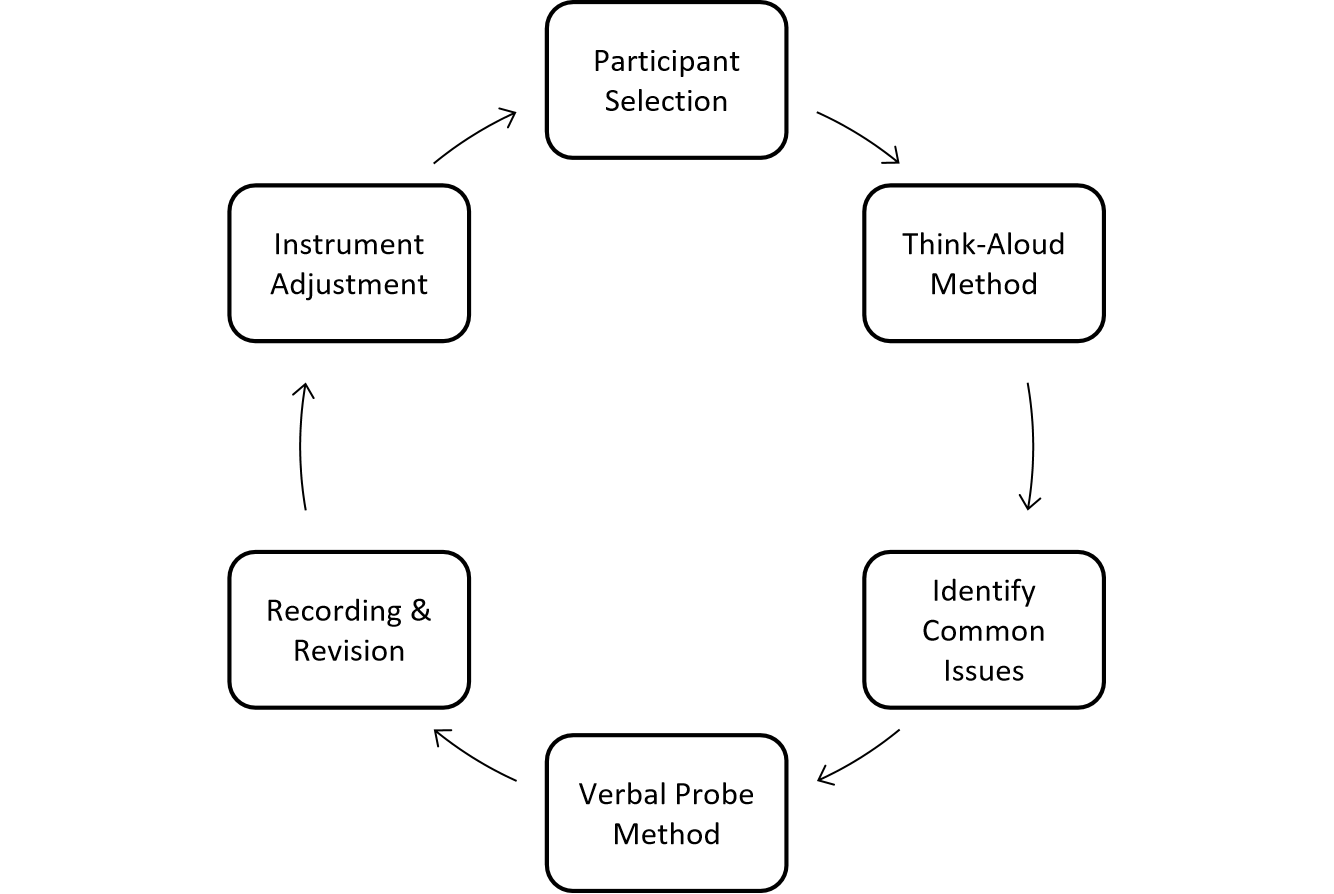

Supplement: Supplementary file 2 [file Supplementary_File_2.docx]
