# Supplementary material for: Translation, cultural adaptation, and psychometric testing of the measure for unfinished care among nursing assistants in long-term care homes in China
Source: Front Public Health. 2026 Apr 16;14:1829774. doi: 10.3389/fpubh.2026.1829774 (PMC13130219; doi:10.3389/fpubh.2026.1829774)
Supplement: Supplementary file 3 [file Table_1.docx]

Supplementary table 1. Prompts for ChatGPT 4.0 during initial and back translation.

| Stages | Prompts for ChatGPT |
| --- | --- |
| Initial Translation | You are a graduate student majoring in English translation. Help me translate the scale in English into Chinese, and keep the original intention unchanged. |
| Back Translation | You are an English teacher in colleges and universities and have engaged in teaching and translation for 10 years. Help me translate the Chinese scale into English, and keep the original intention unchanged. |
