# Supplementary material for: Translation, cultural adaptation, and psychometric testing of the measure for unfinished care among nursing assistants in long-term care homes in China
Source: Front Public Health. 2026 Apr 16;14:1829774. doi: 10.3389/fpubh.2026.1829774 (PMC13130219; doi:10.3389/fpubh.2026.1829774)
Supplement: Supplementary file 4 [file Table_2.docx]

Supplementary table 2. Interview outline of cognitive debriefings

| Interview Procedure | Interview Questions |
| --- | --- |
| Think-Aloud Method | Which question on the scale did you find confusing |
|  | Which question do you find inappropriate or inapplicable |
| Verbal probe method | How do you understand this question |
|  | Do you think the scenario mentioned in this question is something you might encounter in your daily work |
|  | Can you answer this question easily |
|  | Do you think these options are suitable for expressing your opinion |
