# Supplementary material for: Translation, cultural adaptation, and psychometric testing of the measure for unfinished care among nursing assistants in long-term care homes in China
Source: Front Public Health. 2026 Apr 16;14:1829774. doi: 10.3389/fpubh.2026.1829774 (PMC13130219; doi:10.3389/fpubh.2026.1829774)
Supplement: Supplementary file 5 [file Table_3.docx]

Supplementary table 3. Demographic information for nursing assistants participating in cognitive interviews

| Gender | Age, y | Education | Work experience | Type of work |
| --- | --- | --- | --- | --- |
| Female | 45-55 | Junior high school | More than 5 years | Full-time job |
| Female | 45-55 | Junior high school | Greater than 2 to 5 years | Full-time job |
| Female | 45-55 | Junior high school | More than 5 years | Full-time job |
| Female | 45-55 | Junior high school | More than 5 years | Full-time job |
| Female | 45-55 | Junior high school | More than 5 years | Full-time job |
| Female | 45-55 | Secondary school | Not more than 6 months | Full-time job |
| Female | 55-64 | High school | More than 5 years | Full-time job |
| Female | 45-55 | Junior high school | Greater than 2 to 5 years | Full-time job |
