# Supplementary material for: Translation, cultural adaptation, and psychometric testing of the measure for unfinished care among nursing assistants in long-term care homes in China
Source: Front Public Health. 2026 Apr 16;14:1829774. doi: 10.3389/fpubh.2026.1829774 (PMC13130219; doi:10.3389/fpubh.2026.1829774)
Supplement: Supplementary file 6 [file Table_4.docx]

Supplementary table 4. Examples of identified challenges and strategies during translation and cultural adaptation with the Basel Extent of Rationing of Nursing Care for LTC homes instrument

| Challenges | Illustrative Example  (Original item & Initial translation) | Issue identified | Adaptation to the final Chinese version and rationale |
| --- | --- | --- | --- |
| Differences in syntax and idiomaticity between English and Chinese | Item 4:"You could not provide patient(s) food if they are hungry at times other than the regular meals."→First Translation:"如果机构里的老人在非正常用餐时间感到饥饿，您无法提供食物给他们."(Direct English translation: "If residents in the facility feel hungry outside of regular meal times, you are not permitted to provide them with food") | Nursing assistants reported phrasing cumbersome, unnatural in Chinese syntax | Revised to:"在非正常用餐时间，你未能给饥饿的老人提供食物." (Direct English translation: "During irregular mealtimes, you could not provide food to older adults who were hungry")  Concise and consistent with Chinese expression conventions |
| The scenarios mentioned in the original scale do not fit with the context in local LTC homes | Items such as "go for a walk," "shopping," "cooking." | Activities uncommon in Chinese LTC homes | Revised to culturally adequate equivalents: "机构内散步"(walking within the facility), "机构外买东西"(buying items outside the facility), "机构内做操" (exercise within the facility) |
| Different English expressions had multiple interpretations in Chinese | Phrases"could not"/"unable to"→ First Translation: "未能(failed)/无法(unable to)" | Different levels of formality & tone; "未能(failed)" too colloquial | Final version selected "无法(unable to)" to ensure accuracy and formal clarity |
